# The contribution of ocular residual astigmatism to anterior corneal astigmatism in refractive astigmatism eyes

**DOI:** 10.1038/s41598-020-80106-6

**Published:** 2021-01-13

**Authors:** Jian Lin

**Affiliations:** Lianyungang Maternal and Child Health Hospital, Lianyungang, 222000 Jiangsu China

**Keywords:** Corneal diseases, Refractive errors

## Abstract

To determine the distribution of ocular residual astigmatism (ORA) in astigmatic eyes and the influence on the anterior corneal (ACA) and refractive astigmatism (RA). A total of 165 children met the inclusion criteria. Right eyes’ data were analyzed. Using Thibos vector analysis to calculate ORA. Spearman correlation analysis was used to obtain the correlation between the magnitude of ORA, ACA and RA. The median magnitude of ORA in astigmatic eyes was 0.57 D, with interquartile range was 0.42 D. And they were main against-the-rule (57.6–75.8%) and oblique astigmatism (13.9–34.5%) ORA. The ORA in 140 eyes (84.8%) acted as an offset to ACA, meanwhile, 25 eyes (15.2%) superimposed it. About 98% (97.9–98.4%) against-the-rule and 75% (73.9–82.5%) oblique ORA counteracted ACA, nevertheless, all with-the-rule ORA had a superimposed effect on ACA. For with-the-rule ACA, about 86% (85.4–85.9%) ORA worked to offset it. There was statistically correlations between ORA and ACA (*r* = 0.17, *P* = 0.03), ORA and RA (*r* = − 0.27, *P* = 0.001). The magnitude of ocular residual astigmatism was relatively small in children’s astigmatic eyes. Both against-the-rule and oblique ORA can counteract with-the-rule ACA.

## Introduction

Half a century ago, Duke-Elder^1^ put forward the concept of residual astigmatism. Twenty three years ago, based on the term residual astigmatism, Alpins^2^ proposed the parameter ocular residual astigmatism (ORA) to express the discrepancy between corneal and refractive astigmatism (RA). Scholars had agreed that ORA is defined the vectorial difference between RA (calculated to the corneal plane) and anterior corneal astigmatism (ACA) on doubled-angle polar coordinates^2–7^. This parameter not only quantifies the astigmatism stemming from the crystalline lens and the posterior corneal surface, but also contemplates the perceptual contribution^3–5^. More concretely, ACA was calculated based on simulated keratometry (SimK) value, the difference in power between the steep and flat meridians, in 3 mm optical zone of the cornea. RA is the total ocular astigmatism obtained by phoropter or retinoscopy.

Astigmatism is a common refractive anomaly that results in reduced visual acuity and causes various symptoms like glare, monocular diplopia, asthenopia, and distortion^8^. It is more difficult to treat compared to other refractive errors. Unfortunately, it was prevails in most human eyes. Previous studies have reported the prevalence of astigmatism from 11.3% up to 70%^9–11^. With the advent of laser techniques, lots of devices were developed to correct the astigmatic refractive errors. A growing number of studies have found that lower efficiency of astigmatism correction by laser systems is mainly due to the eye's internal optics, crystalline lens is the largest component of it. Specifically, Qian et al.^12^ found that LASIK was more than twice as effective in ORA/RA < 1.0 eyes as in ORA/RA ≥ 1.0 eyes, the mean index of success was 0.88 and 0.32 respectively. Wallerstein^13^ obtained the similar outcomes by analysis Topography-Guided Ablation and conventional LASIK.

Previous studies had shown that ORA were mainly against-the-rule astigmatism, and provided a negative effect for ACA which were normally with-the-rule in eyes^14,15^. But no articles have examined this role in depth. The aim of the current study was to determine the specific effects of ORA to ACA.

## Materials and methods

This study followed the tenets of the Declaration of Helsinki and was approved by Lianyungang Maternal and Child Health Hospital review board. Informed consent was obtained from each subject’ parents after explanation of the nature of the study.

### Participants selection

We conducted a cross-sectional study of children diagnosed with RA (spectacle plane) ≥ 1.00D who presented at the outpatient ophthalmology clinics at Lianyungang Maternal and Child Health Hospital during June 2019 to September 2019. Excluding organic diseases of eyes such as cataract, glaucoma, keratoconus, irregular astigmatism, nystagmus and children with strabismus, a total of 165 children met the inclusion criteria: 83 females and 82 males. The mean age was 4.8 ± 1.1 years. Only right eyes data are taken for analysis.

### Examination protocol and collect parameters

After one drop of topical anaesthetic agent (Alcaine, Alcon), cycloplegia process was performed by two drops of 1% cyclopentolate (Alcon) and one drop of Mydrin P (Santen, Japan), once every 5–8 minutes^16^. To press the lacrimal sac for 3 min after each time, and wait for at least 30 min after three times until the pupillary reaction to light disappeared or only the weak light reflex remained. If the pupillary light reflex was still present or the pupil size was less than 6.0 mm, a third drop of cyclopentolate was instilled. Objective refraction (NIDEK Automated Refractor/Keratometer (ARK-1) , NIDEK, Japan) were completed to analyze the refractive status and corneal curvature of the participants under cycloplegic conditions^11^. Multiplying the curvature by 0.3375 to calculate the corneal power. Measured two times, took the mean value of the results with confidence ≥ 8. RA is the cylindrical part of refractive error at the corneal plane. ACA is the difference in power between the steep and flat meridians. The NIDEK ARK-1 measured the radius of corneal curvature within 3 mm optical zone of the cornea by Placido ring principle.

### Data analysis and calculations

In mathematics and physics, the positive and negative of a vector is determined by its direction, and its size is only positive. So both RA and ACA are converted into the positive-cylinder notation before calculation.

RA and ACA were transformed into power vector components using:

$$ \begin{aligned} J_{0(RA)} & = - \frac{RA}{2} \times COS\left( {2\beta_{RA} } \right),\quad J_{45(RA)} = - \frac{RA}{2} \times SIN\left( {2\beta_{RA} } \right) \\ J_{0(ACA)} & = - \frac{ACA}{2} \times COS\left( {2\beta_{ACA} } \right)\quad J_{45(ACA)} = - \frac{ACA}{2} \times SIN\left( {2\beta_{ACA} } \right) \\ \end{aligned} $$

where J_0_ and J_45_ were the horizontal/vertical and oblique components of astigmatism, respectively, and β was the positive-cylinder axis.

The components of ORA were determined as:

$$ J_{0(ORA)} = J_{0(RA)} - J_{0(ACA)} ,\quad J_{45(ORA)} = J_{45(RA)} - J_{45(ACA)} $$

So the magnitude and axis (β _ORA_) of ORA were calculated as:

$$ ORA = 2\sqrt {J_{0(ORA)}^{2} + J_{45(ORA)}^{2} } ,\quad 2\beta_{ORA} = a\tan \left( {\frac{{J_{45(ORA)} }}{{J_{0(ORA)} }}} \right) $$

The unique β_ORA_ was determined according to the vector relationship of ORA, ACA and RA in the double angle vector diagram. Two examples were gave to illustrate this point.

**Figure 1:**
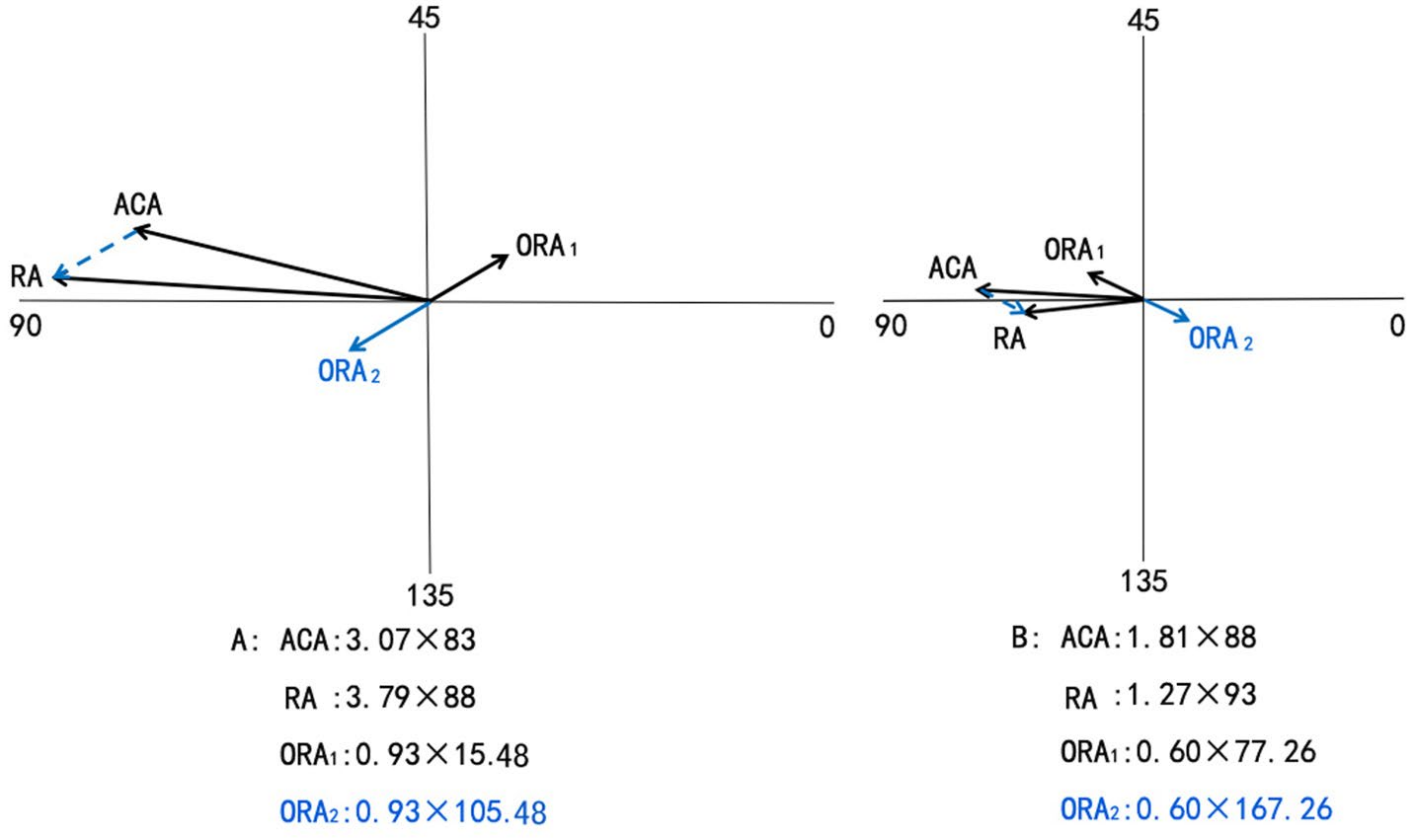
The calculation process of ORA. *ORA*_*1*_: The erroneous ORA. *ORA*_*2*_: The actual ORA.

*Case 1*: ACA:3.07 × 83, RA:3.79 × 88, so $\tan \left( {2\beta_{ORA} } \right) = \left( {\frac{{J_{45(RA)} - J_{45(ACA)} }}{{J_{0(RA)} - J_{0(ACA)} }}} \right) = 0.60$. 2β_ORA_ is 30.96° or 210.96°calculated by arc tangent. According to the vector relation of ORA, ACA, RA in the double angle vector diagram, we can make certain 2β_ORA_ is 210.96°, so β_ORA_ is 105.48° (Fig. 1A).

*Case 2*: ACA:1.81 × 88, RA :1.27 × 93, so $\tan \left( {2\beta_{ORA} } \right) = \left( {\frac{{J_{45(RA)} - J_{45(ACA)} }}{{J_{0(RA)} - J_{0(ACA)} }}} \right) = - 0.48$, arctan (− 0.48) = − 25.49°, so 2β_ORA_ is 154.51° or 334.51°. According to the vector relation of ORA, ACA, RA in the double angle vector diagram, we can make certain 2β_ORA_ is 334.51°, not 154.51°. So β_ORA_ is 167.26° (Fig. 1B).

The orientation of astigmatism was grouped for with-the-rule (ie, correcting axis of positive cylinders at or near 90°), against-the-rule (ie, correcting axis of positive cylinders at or near 180°) and oblique astigmatism. There were different classification standards, including ± 15°, ± 20° and ± 30°.

### Statistical methods

SPSS statistics software package version 17.0 for Windows (IBM, Armonk, NY, USA) was used for the statistical analysis and calculations. Normality of all data samples was checked by means of the Kolmogorov–Smirnov test. The magnitude of ACA, RA and ORA all were non-normally distributed. The non-normality measurement data were expressed as median value and interquartile distance. Correlation coefficients (Pearson or Spearman depending if normality condition could be assumed) were used to assess the correlation of ORA and ACA, RA. Correlations were considered to be statistically significant when the associated *p*-value was < 0.05.

## Results

**Figure 2:**
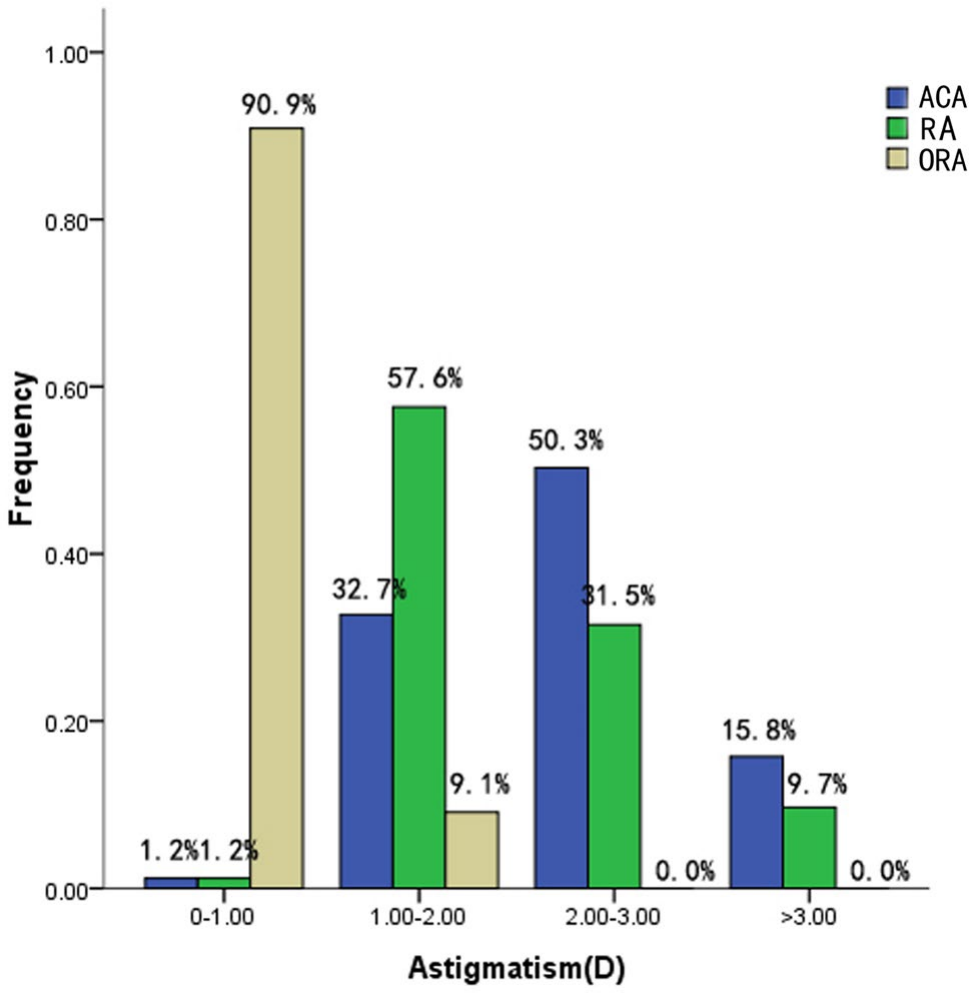
Distributions of the magnitude of RA, ACA, and ORA at corneal plane.

The study included a total of 165 right eyes from 165 children with a mean age of 4.8 years old (SD: 1.1; range: 3–6 years). They were 83 females and 82 males. 73 (44.2%) eyes had compound hyperopic astigmatism, 66 (40.0%) eyes shown mixed astigmatism, 10 (6.1%) eyes displayed compound myopic astigmatism, 9 (5.5%) were simple hyperopic astigmatism, and 7 (4.2%) proved simple myopia astigmatism. At the corneal plane, mean cycloplegic spherical equivalent was 0.92 D (range − 3.81 D to + 6.77 D). The ACA ranged 0.32 D to 4.83 D (median value was 2.26 D, interquartile range (IQR) was 0.89 D). The RA ranged 0.91 D to 4.68 D (median value was 1.79 D, IQR was 1.09 D). The ORA ranged 0.01 D to 1.87 D (median value was 0.57 D, IQR was 0.42 D). ACA was mainly concentrated above 2.0 D, the majority of RA ranged 1.0 D to 2.0 D. The ORA of 150 refractive astigmatism eyes (90.9%) was less than 1.0 D. The ORA of other eyes (9.1%) ranged 1 D to 2 D. Figure 2 shows the distributions of the magnitude at corneal plane.

With different limits of astigmatism axes classification, including ± 15°, ± 20° and ± 30°, 157 (95.2%) to 163 (98.8%) eyes had with-the-rule ACA. 1(0.6%) to 2(1.2%) eyes had against-the-rule ACA. 0 (0.0%) to 7 (4.2%) eyes had oblique ACA. 147 (89.1%) to 163 (98.8%) eyes were with-the-rule RA. 2 eyes (1.2%) were against-the-rule RA. 0 (0.0%) to 16 eyes (9.7%) were oblique RA. 95 (57.6%) to 125 (75.8%) eyes shown against-the-rule ORA, 13 (7.9%) to 17 (10.3%) eyes shown with-the-rule ORA, 57 (34.5%) to 23 (13.9%) eyes shown oblique ORA. Data materials were shown in Table 1.

**Table 1:** The distribution of RA, ACA, ORA with different classification of astigmatism axes in current study.

	RA			ACA			ORA		
	WTR	ATR	Oblique	WTR	ATR	Oblique	WTR	ATR	Oblique
± 15°	147 (89.1%)	2 (1.2%)	16 (9.7%)	157 (95.2%)	1 (0.6%)	7 (4.2%)	13 (7.9%)	95 (57.6%)	57 (34.5%)
± 20°	158 (95.8%)	2 (1.2%)	5 (3.0%)	161 (97.6%)	1 (0.6%)	3 (1.8%)	14 (8.5%)	115 (69.7%)	36 (21.8%)
± 30°	163 (98.8%)	2 (1.2%)	0 (0.0%)	163 (98.8%)	2 (1.2%)	0 (0.0%)	17 (10.3%)	125 (75.8%)	23 (13.9%)

### ORA outcomes

**Figure 3:**
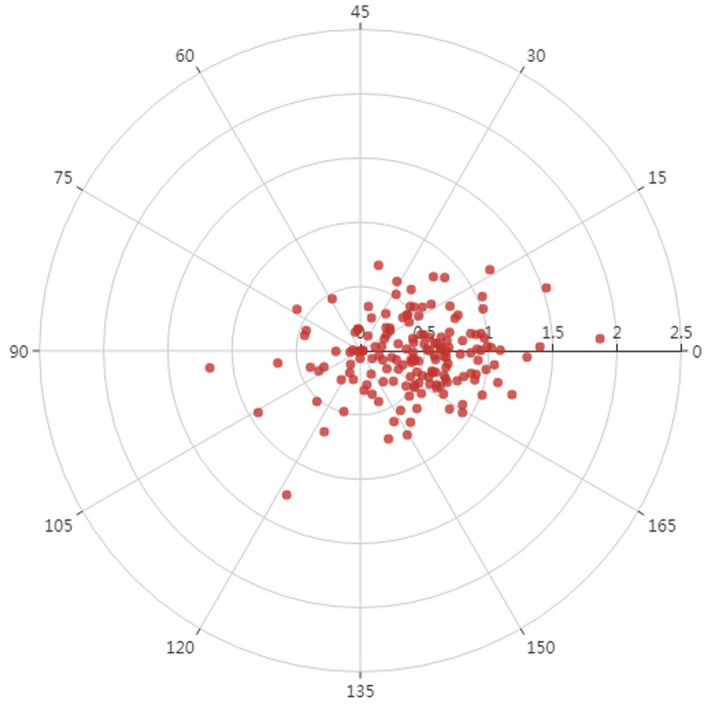
The distribution of ORA obtained in the refractive astigmatism eyes on double angle vector diagram.

Figure 3 shows the distribution of the ORA outcomes obtained in the sample of refractive astigmatism eyes on double angle vector diagram. Median magnitude of ORA was 0.57 D (IQR: 0.42 D), ranged 0.01 D to 1.87 D. There was statistically significant correlations between the magnitude of ORA and ACA (*r* = 0.17, *P* = 0.03), ORA and RA (*r* = − 0.27, *P* = 0.001).

### The influence of ORA on ACA

In mathematics and physics fields, it was considered that the angles between two vectors cancel out each other when they were greater than 90° and overlap each other when they were less than 90°. Since the vector angle was twice as large as the axis, therefore, when the axial difference between ORA and ACA was greater than 45°, ORA will canceled out ACA. When the difference was less than 45°, ORA had the superposition effect on ACA. In this group of data, the ORA of 140 eyes (84.8%) counteracted the ACA, 25 eyes (15.2%) shown superimposed effect on ACA. The influence of ORA on ACA was analyzed and found that, with different classification standards, about 98% (from 93/95 to 123/125) against-the-rule and 75% (from 17/23 to 47/57) oblique ORA counteracted ACA, nevertheless, all with-the-rule ORA had a superimposed effect on ACA (Table 2).

**Table 2:** The influence of ORA on ACA with different classification standards.

	Effect of counteraction (N = 140)			Effect of superposition (N = 25)		
	WTR	ATR	Oblique	WTR	ATR	Oblique
** ± 15°**						
ACA	134	0	6	23	1	1
ORA	0	93	47	13	2	10
** ± 20°**						
ACA	138	0	2	23	1	1
ORA	0	113	27	14	2	9
** ± 30°**						
ACA	140	0	0	23	2	0
ORA	0	123	17	17	2	6

For with-the-rule ACA, about 86% (from 134/157 to 140/163) ORA worked to offset it. All with-the-rule ORA played a superimposed role on with-the-rule ACA, and all against-the-rule ORA canceled with-the-rule ACA, with approximately 76% (17/23–44/54) of oblique ORA cancelling with-the-rule ACA (Table 3).

**Table 3:** The effect of ORA on with-the-rule ACA with different classification standards.

WTR ACA ORA	Effect of counteraction			Effect of superposition		
	WTR	ATR	Oblique	WTR	ATR	Oblique
± 15°(N = 157)	0	90	44	13	0	10
± 20°(N = 161)	0	113	25	14	0	9
± 30°(N = 163)	0	123	17	17	0	6

## Discussion

The ORA was defined as that cannot be attributed to the anterior corneal surface astigmatism, including posterior corneal astigmatism, lenticular astigmatism and retinal astigmatism^12,17^. It is also designated with other terminologies, such as lenticular astigmatism^18^. The ORA were main against-the-rule astigmatism in human eyes. So most clinicians suggested that the ORA provided a compensatory effect for ACA, which was normally with-the-rule in the normal population^14,15^. In this group of data, 95 (57.6%) to 125 (75.8%) eyes shown against-the-rule ORA with different limits of astigmatism axes classification. ORA of 140 eyes (84.8%) counteracted the ACA, 25 eyes (15.2%) shown superimposed effect on ACA. However, the specific impact of ORA on ACA remains unclear. Duke-Elder^19^ considered that lenticular astigmatism can counteract with-the-rule corneal astigmatism and superimpose against-the-rule corneal astigmatism, however, the rule of oblique astigmatism is not clear. This paper describes the contribution of ORA to ACA, we found that, with different classification standards, about 98% (from 93/95 to 123/125) against-the-rule and 75% (from 17/23 to 47/57) oblique ORA counteracted ACA, nevertheless, all with-the-rule ORA had a superimposed effect on ACA (Table 2). For with-the-rule ACA, about 86% (from 134/157 to 140/163) ORA worked to offset it. All with-the-rule ORA played a superimposed role on with-the-rule ACA, and all against-the-rule ORA canceled with-the-rule ACA, with approximately 76% (17/23–44/54) of oblique ORA cancelling with-the-rule ACA (Table 3).

The magnitude of ORA in children refractive astigmatism eyes range from 0.01D to 1.87D (median value: 0.57D, IQR : 0.42 D). This value is less than the results in normal adult eyes from other studies. Piñero et al.^20^ found the magnitude of ORA in normal adult eyes ranged between 0.07 and 2.58 D, with a mean value of 0.79 D (SD: 0.43). Plech et al.^21^ found it ranged from 0.13 to 2.88 D , with 0.78 ± 0.61 D. The difference of RA may be the main reason for this result. Spearman correlation analysis showed that there was statistically correlations between the magnitude of ORA and ACA (*r* = 0.17, *P* = 0.03), ORA and RA (*r* = -0.27, *P* = 0.001). This implies that ORA is more strongly but negatively correlated with RA than ACA, i.e., the greater the RA are, the smaller the ORA are. In addition, the magnitude of ORA also determines the ability of its influence on ACA. A study had shown that the prevalence of ACA is significantly higher than RA^16^. This findings suggested that low amounts of ORA counteracts too little ACA and eventually leads to too much RA. One recent study with comparison on the ORA between myopes and non-myopes found that ORA may decrease the contribution of ACA to RA in eyes with small amounts of ACA^22^.

To investigate the contribution of ORA to ACA is also crucially in laser surgery. The ORA had been shown to be ubiquitous after keratorefractive procedures and to be in relation with postoperative astigmatism^2,13,17^. Therefore, the contribution of ORA to ACA should not be ignored in corneal laser surgery. This suggests that before corneal refractive surgery, the magnitude and axis of ORA should be calculated first. For patients with high ORA / RA, the purpose of laser ablation is to retain corneal astigmatism with the same magnitude as ORA and the axial difference of 90°, which may be more beneficial. When large ORA existed, Alpins^23^ suggested to leave 60% of ORA on the cornea (rather than the customary 100%) and 40% in the wavefront refraction 2nd-order component (instead of the usual 0%). This treatment produced a greater reduction in ACA and better visual outcomes than the conventional treatment. It is generally known that ablation on the steepest meridian of ACA can reduce the depth of ablation. If the ORA is decomposed to the steepest meridian of ACA and its vertical meridian, the amount of surgery can be redesigned, which may benefit the patients with thin cornea.

In conclusion, in this study we found the magnitude of ORA was relatively small in children’s astigmatic eyes. Most of ORA (84.8% in this study) counteracted the anterior corneal astigmatism. There was a significant correlation between ACA, RA and ORA.

### Ethics approval and consent to participate

The study protocol was approved by the Lianyungang Maternal and Child Health Hospital, adhered to the tenets of the Declaration of Helsinki.
