# Author Correction: The contribution of ocular residual astigmatism to anterior corneal astigmatism in refractive astigmatism eyes

**DOI:** 10.1038/s41598-021-93185-w

**Published:** 2021-07-01

**Authors:** Jian Lin

**Affiliations:** Lianyungang Maternal and Child Health Hospital, Lianyungang, 222000 Jiangsu China

Correction to: *Scientific Reports* 10.1038/s41598-020-80106-6, published online 13 January 2021

This Article contained errors. In the originally published version, the following statement in the Discussion was inaccurate:

“Qian et al.^12^ found that LASIK was more than twice as effective in low ORA group (ORA/RA < 1.0) as in high ORA (ORA/RA ≥ 1.0).”

This was now corrected and reads:

“Qian et al.^12^ found that LASIK was more than twice as effective in ORA/RA < 1.0 eyes as in ORA/RA ≥ 1.0 eyes.”

This change does not affect the conclusions of the Article. The original Article has been corrected.

